# Mechanism for enhancing the growth of mung bean seedlings under simulated microgravity

**DOI:** 10.1038/s41526-021-00156-6

**Published:** 2021-07-15

**Authors:** Shusaku Nakajima, Masayasu Nagata, Akifumi Ikehata

**Affiliations:** 1 Graduate School of Agricultural Science, Kobe University, Kobe, Japan; 2 Food Research Institute, National Agriculture and Food Research Organization, Tsukuba, Japan

**Keywords:** Agriculture, Plant sciences

## Abstract

To elucidate a mechanism for enhancing mung bean seedlings’ growth under microgravity conditions, we measured growth, gene expression, and enzyme activity under clinorotation (20 rpm), and compared data obtained to those grown under normal gravity conditions (control). An increase in fresh weight, water content, and lengths were observed in the clinostat seedlings, compared to those of the control seedlings. Real-time PCR showed that aquaporin expression and the amylase gene were upregulated under clinorotation. Additionally, seedlings under clinorotation exhibited a significantly higher amylase activity. Near-infrared image showed that there was no restriction of water evaporation from the seedlings under clinorotation. Therefore, these results indicate that simulated microgravity could induce water uptake, resulting in enhanced amylase activity and seedling growth. Upregulated aquaporin expression could be the first trigger for enhanced growth under clinorotation. We speculated that the seedlings under clinorotation do not use energy against gravitational force and consumed surplus energy for enhanced growth.

## Introduction

Space, which is the final frontier for humans, can be a new source of water, minerals, and human habitation^1,2^. However, an abnormal space environment, notably microgravity, causes severe unfavorable effects, such as bone loss, cardiovascular disease, lung deformation, and DNA damage^3–7^. Since the phytochemical components of fresh vegetables contribute to reducing these risks, similar cultivation systems here on the Earth are essential for future long-duration space missions. Microgravity is a unique environment that induces physical and physiological changes in plants, and a comprehensive understanding of plant growth and development under microgravity is required for space agriculture.

Because of limited access to spaceflight, rotation devices, such as a clinostat (CL) and Radom Positioning Machine (RPM), have been used to generate microgravity here on the Earth. Although simulated microgravity is not the same as real microgravity in space^8,9^, these devices can cultivate plants repeatedly at a low cost. Recent studies have demonstrated that a fast rotating system with a small radius around a rotating axis can provide better microgravity than slow rotating system and RPM^10–12^. Therefore, most researchers cultivating plants under clinorotation employed fast rotation speed 20–60 rpm, depending on sample size^13,14^.

Previous experiments performed in space and by ground-based simulations have revealed that microgravity can enhance growth and phytochemical properties at the early developmental stage of specific plants. For instance, *Arabidopsis* grown in space developed longer seedlings and larger leaves, compared to the ground control^15,16^. Root elongation was reported in sweet potato grown in space, *Brassica napus* L. (1 rpm) and mung bean (2 rpm) grown under clinorotation^17–19^. Additionally, a higher accumulation of phytochemical components was found in *Brassica rapa* L. and soybean seedlings during spaceflight^20,21^. There are reports on the enhanced antioxidant activity of mung bean seedlings grown under clinorotation (2 rpm) and antidiabetic properties of wheatgrass grown under RPM^19,22^. The rapid growth will contribute to shorter cultivation and enhanced phytochemical properties, as a countermeasure against the dangerous space environment. However, little is known about the mechanism of microgravity and its positive effects on the early developmental stage of seedlings.

Seed germination is triggered by water uptake, after a lag phase, followed by radicle elongation^23^. Subsequently, reserve energy accumulated in seeds is hydrolyzed by specific enzymes for seedling growth. Aquaporin is an intrinsic protein that governs water transport in various processes, including germination^24,25^. Furthermore, in the case of legumes, the degradation of accumulated starch in seeds begins with the synthesis of α-amylase activated by absorbed water, and the conversion of starch to oligosaccharides, which is further hydrolyzed to maltose by β-amylase^26^. Maltose is then hydrolyzed by α-glucosidase with the release of glucose, which plays a significant role in fueling plant growth and development before the leaves can begin to photosynthesis. Thus, we hypothesized that microgravity affects water uptake and energy hydrolysis involved in germination and early growth.

This study aims to elucidate the cause of enhanced growth under microgravity conditions. Therefore, we cultivated mung bean under clinorotation, and measured aquaporin and amylase activity involved in water uptake and energy hydrolysis during germination. Data for enhanced growth due to aquaporin and amylase activity of seedlings grown under clinorotation are shown in the Results and Discussion section in this article.

## Results

### Growth of seedlings

The growth of mung bean seedlings under the control and clinorotation is shown in Table 1. The fresh weight and water content of seedlings under grown clinorotation were significantly higher than those grown under the control conditions. Additionally, the seedlings grown under clinorotation developed a significantly longer shoot and root than those grown under the control conditions. Similar results on the positive influence of microgravity in spaceflight and ground-based simulations were reported, as described in the Introduction.

**Table 1:** Growth of seedlings (*n* = 30).

Growth condition	Fresh weight (mg)	Water content (%)	Length (cm)	
			Stem	Root
Clinorotation	332.33 ± 8.53*	85.49 ± 0.44*	4.43 ± 0.21*	5.05 ± 0.36*
Control	298.67 ± 9.64	83.66 ± 0.45	3.35 ± 0.25	3.50 ± 0.31

Asterisks indicate significant difference between control and clinorotation.

### Water distribution

**Fig. 1:**
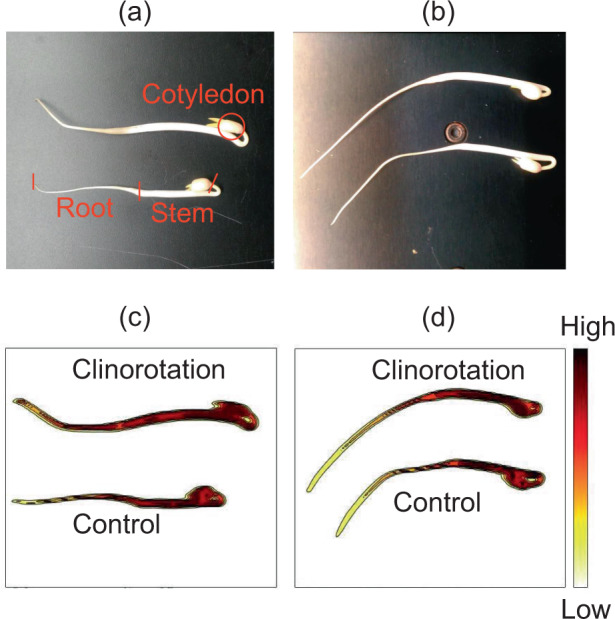
Mung bean seedlings grown under clinorotaion and control. **a**, **b** Visible images of seedlings. The cotyledon, stem, and root sections were marked by red. (**c**, **d**) The NIR images of same seedlings. The color bar indicates second derivative intensity at 1418 nm. The upper seedlings were grown under clinorotation and bottom seedlings were grown under the control conditions.

To further examine the water state in seedlings grown under clinorotation, we monitored water distribution using a near-infrared (NIR) imaging system. Fig. 1 indicated both visible and NIR images of the control and CL seedlings. Although water content significantly increased under clinorotation (Table 1), there was no specific change in water distribution among seedlings grown under control and clinorotation. Water loss was suppressed in harvested mung bean seedlings under 3D-CL (2–4 rpm) compared to normal gravity conditions^27^, but no such feature was observed in the growth stage of the seedlings in this study. Stem elongation resulted in a higher water content under clinorotation than roots as seen in the NIR images.

### Aquaporin gene expression

We measured the gene expression of plasma membrane intrinsic proteins (*PIP*) and tonoplast intrinsic proteins (*TIP*) in the roots (Table 2). In mung bean roots, *PIP1-2* and *PIP2-1* expressions were higher than other aquaporins. While no changes in *PIP2-1* and *PIP2-2* expression, the *PIP1-2*, *PIP1-4*, *TIP1-1*, and *TIP1-3* were significantly activated in the seedlings grown under clinorotation. The increase in water content under clinorotation was either due to enhanced water uptake or reduced evaporation. If the latter is the case, water should be accumulated in the seedlings, resulting in water distribution changes under clinorotation. However, that was not the case based on the NIR image (Fig. 1b). Thus, upregulated aquaporin expressions indicate that higher water content in seedlings under clinorotation could be due to enhanced water uptake ability.

**Table 2:** Aquaporin gene expression in roots (*n* = 10).

Growth condition	Relative gene expression					
	*PIP1-2*	*PIP1-4*	*PIP2-1*	*PIP2-2*	*TIP1-1*	*TIP1-3*
Clinorotation	1.54 ± 0.07*	7.31 ± 0.71 (×10^−3^)*	2.32 ± 0.10	3.10 ± 0.32 (×10^−3^)	4.55 ± 0.16 (×10^−3^)*	1.97 ± 0.08 (×10^−3^)*
Control	1.26 ± 0.07	4.49 ± 0.85 (×10^−3^)	2.07 ± 0.11	2.45 ± 0.27 (×10^−3^)	3.26 ± 0.41 (×10^−3^)	1.50 ± 0.20 (×10^−3^)

Asterisks indicate significant difference between control and clinorotation.

### Amylase gene expression and activity

**Fig. 2:**
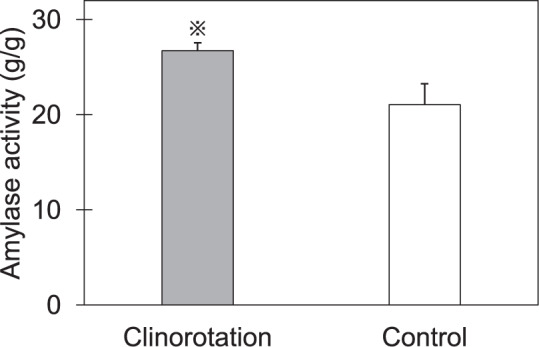
Amylase activity in cotyledons (*n* = 10). Asterisks indicate significant difference between control and clinorotation.

The amylase gene expression and amylase activity in cotyledons are shown in Table 3 and Fig. 2, respectively. The expression of four amylase genes encoding α-amylase and β-amylase were significantly higher in the cotyledons grown under clinorotation. Additionally, the cotyledons of seedlings under clinorotation exhibited 27% higher amylase activity. These results showed that starch hydrolyzed enzymes in cotyledons were activated under clinorotation.

**Table 3:** Amylase gene expression in cotyledons (*n* = 10).

Growth condition	Relative gene expression (×10^−2^)			
	*α-amylase*	*α-amylase 2*	*β-amylase*	*β-amylase 1*
Clinorotation	11.24 ± 1.98*	6.74 ± 1.38*	4.37 ± 0.79*	29.15 ± 3.70*
Control	3.24 ± 0.72	1.81 ± 0.36	0.70 ± 0.35	9.19 ± 2.00

Asterisks indicate significant difference between control and clinorotation.

## Discussion

In this study, we showed that simulated microgravity generated by clinorotation promotes gene expression and hydrolysis enzyme activity involved in the germination process, as hypothesized. Notably, the upregulated aquaporin expression could be the trigger for enhanced growth under microgravity because water uptake is the first step in germination and subsequent growth. Terrestrial plants have adapted to the constant gravitational condition here on the Earth after evolution from the sea and must consume energy to maintain homeostasis against gravitational force^28^. In contrast, plants grown under microgravity do not need such energy, as shown in previous reports. Protoplasts isolated from tobacco used less metabolic energy for regeneration during spaceflight^29^. Soleimani et al.^14^ also observed an increase in growth and metabolism of tobacco cells grown under clinorotation (20 rpm), which suggests that an energy-saving process occurs under simulated microgravity. Apart from other organisms, the use of less energy was observed in human lung cells cultivated in space^30^. Therefore, we speculated that the mung bean seedlings grown under clinorotation exhibited a similar energy-saving process and used its surplus energy for upregulating aquaporin expression.

Aquaporin expression and water flow under microgravity are significant subjects of discussion in space experiments. Jing et al.^31^ observed a similar upregulation of aquaporins in rice calli. Wang et al.^32^ also observed an enhanced guttation in rice seedlings, therefore suggesting that water uptake and transport are easier under microgravity. These data from previous studies agree with data obtained in this study on the upregulation of aquaporin and higher water content under clinorotation. Although the detailed roles of aquaporins are still underdetermined, previous plant studies have revealed that *PIP1* and *TIP1*, which are upregulated under clinorotation (Table 2), are involved in cell division and tissue elongation after germination. For example, in the positive control using an aquaporin activator, increased water content and elongation were observed in various germinating seedlings^33,34^. Alternatively, in the negative control using an aquaporin inhibitor, a lower expression of aquaporin resulted in delayed or abnormal growth^25^. These results support the fact that upregulated aquaporin enhances growth under clinorotation.

Since there are a few reports on amylase activity in germinating seedlings grown under microgravity, it is difficult to compare our data with that of previous studies directly. However, α-amylase activity is significantly suppressed in wheat seedlings exposed to hypergravity^35,36^. Generally, the influence of microgravity and hypergravity is opposite, and there is the possibility that amylase activity is promoted in wheat seedlings germinating under microgravity conditions. Since starch is accumulated in wheat seeds as reserve energy like in mung bean, these results suggest that microgravity induces amylase activity in these types of seeds.

**Fig. 3:**
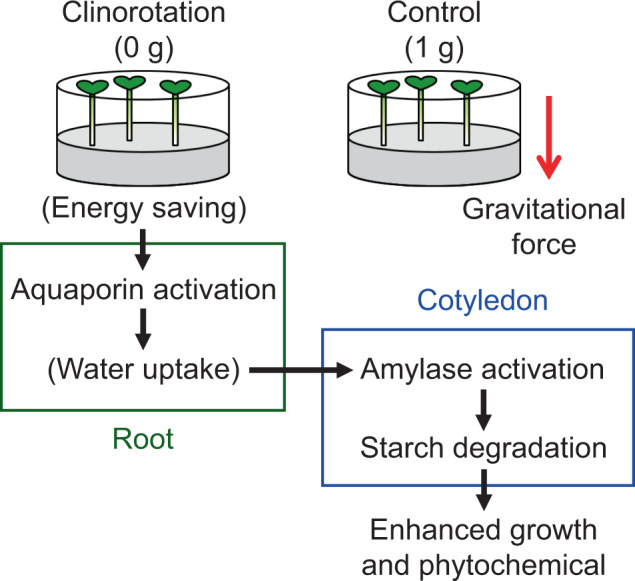
The potential mechanism for a positive growth in mung bean seedling grown under clinorotation obtained in this and our previous study^19^. The green area was observed in roots, whereas the blue area was observed in cotyledons. Parentheses are discussions and not obtained data.

A potential mechanism for enhanced growth and phytochemical properties in mung bean seedlings under clinorotation observed in this, and previous studies^19^ is shown in Fig. 3. Clinorotation first activates aquaporin activity in roots (Table 2) and could promote water uptake ability. The enriched water condition in the seedlings under clinorotation further induces amylase gene expression encoding α- and β-amylase (Table 3), resulting in higher amylase activity in cotyledons (Fig. 2). Our previous study showed that starch accumulated in seeds is rapidly degraded under clinorotation, and the seedlings could have more sugars converted from starch^19^. We believe that these sugars may be involved in the enhanced growth and phytochemical properties under clinorotation. Indeed, upregulated aquaporin expression could be the first step for enhanced growth under clinorotation.

The positive effects of microgravity on the early developmental stage of plants have been reported, but the leading cause for this is still unknown. In contrast, we discovered that simulated microgravity generated by clinorotation activated aquaporin and hydrolyzed enzymes in mung bean seedlings, thereby resulting in enhanced growth. These results strongly recommended that similar plants with enhanced aquaporin and hydrolyzed enzymes under microgravity are suitable for space agriculture. Since the effects of microgravity vary on plant species^37^, further investigation on gene expression in other plants is needed. Additionally, there is a need for further experiments to be conducted in real microgravity in space for a more comprehensive understanding of microgravity effect.

## Methods

### Plant materials and growth conditions

**Fig. 4:**
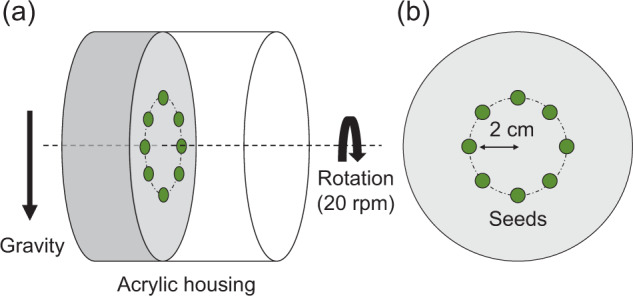
Schematic illustration of clinorotation. **a** Side and **b** front views. Eight mung bean seeds were placed in an acrylic housing at a distance of 2 cm from the axis in a circle and rotated at 20 rpm for 3 d.

Seedlings were rotated under a similar experimental system used in our previous study^19^. The CL consisted of an AC servo motor (SGMAH-A5BAA21, Yasukawa Electric, Japan) and a cylindrical acrylic housing (9.4 cm diameter × 9.0 cm height), which were filled with 0.8% (w/w) agar medium (3.0 cm depth). Eight seeds were germinated and cultivated in the center of the rotation axis within a circle of 2 cm radius. In the CL experiment (Fig. 4), the rotor and seedlings axes were horizontal (i.e., perpendicular to the g-vector) and rotated at 20 rpm. At this rotation speed, centrifugal acceleration was 8.9 × 10^−3^ g. Seedlings grown in the control conditions were cultivated in the housing and placed on the bottom of the same incubator. Seedlings in both growth conditions were cultivated in darkness at 25.0 ± 1.0 °C for 3 d. To obtain fundamental data, fresh weight, length, and water content were measured after 3 d of cultivation. According to a previous study^19^, stem sections between the cotyledon/hypocotyl interface and the hypocotyl/root interface were determined. We also determined root sections between the hypocotyl/root interface and the root tip. In addition, the water content was calculated by gravimetric determination, after seedlings were oven-dried at 80 °C for 3 d.

### NIR imaging

Since water has high absorption bands in the NIR regions, NIR spectra and images can monitor water content in plants^38,39^. To measure water distribution under clinorotation, hyperspectral NIR image was captured by Imspector N17E (Specim, Finland) in the range of 950–1600 nm, according to the previous method^39^ with some modifications. We first measured the intensity of polytetrafluoroethylene (PTFE) reference reflector (Spectralon®, Labsphere, Inc., North Sutton, NH, USA), and obtained reflectance spectra of seedlings. After transforming absorbance spectra, we employed second derivative treatments and the intensities at 1418 nm of each pixel were used for NIR imaging.

### Gene expression analysis

Measurement of gene expression was performed according to the method^40^. Seedlings were immediately frozen in liquid nitrogen after cultivation and stored at −80 °C until use. RNA was extracted using the RNeasy plant Mini Kit (Qiagen, Netherlands). The quality of RNA was measured by a UV spectrophotometer (Nano-200, Medclub Scientific, Taiwan) and cDNA was synthesized according to the method of PrimeScript RT reagent Kit with gDNA Eraser (Takara, Japan). Quantitative PCR was conducted in triplicate using the Thermal Cycler Dice Real Time System TP800 (Takara, Japan), with TB Green Premix Ex Taq II (Tli RNaseH Plus, Takara, Japan) and specific primers (Table 4). We analyzed the expression of four amylase genes in cotyledons, namely *α-amylase*, *α-amylase 2*, *β-amylase*, and *β-amylase 1*, and six aquaporin genes in roots, namely *PIP1-2*, *PIP1-4*, *PIP2-1*, *PIP2-2*, *TIP1-1*, and *TIP1-3*. The expression value of *actin* normalized relative expression values.

**Table 4:** Real-time PCR primer sequences.

Forward primer	Reverse primer	Annotation	Accession number
TTCCTAATAGTTTTCAAACACGCT	TCTCAGACACCAGTTTTGGAAGT	alpha-amylase	XM_014659502
GTGTTGGTGGTAGAAGAAGAAGAGA	GATCGCAAGCTACAACCACCA	alpha-amylase 2	XM_014648970
GTTACAAGCAGCAGGTGTCG	CAGGGCCCTTGGATTCAACT	beta-amylase	XM_022780276
AAGCACCCATAGAGGAAGCG	GGAATTGTTTCCTAGTCATGAGCC	beta-amylase 1	XM_014650101
CCCTTGTCTACTGCACTGCT	GCCCTTGTGAGGGACAACTT	aquaporin PIP1-2	XM_014654123
CGCATGTTCCTCACCTTTGC	GTTGATGCCAGTCCCTGTGA	aquaporin PIP1-4	XM_014658364
GGCGAAGGACGTTGAGGTTA	CATCAATGAGGGGTGCAGGA	aquaporin PIP2-1	XM_014658124
TGGAGCAACCATTGCAGTCTT	GCCTCATCCTCCACTTCAACT	aquaporin PIP2-2	XM_014657769
ACATTTGCTTTCGTGAGCGG	GGCTCCAACGACTAAGCCAA	aquaporin TIP1-1	XM_014666999
ACAATGGACCTGCAACACCT	GAATGGACTGCCCCAACAGA	aquaporin TIP1-3	XM_014641881
TTACAGCATTGGCACCGAGT	GGAGCCTCCAATCCAGACAC	actin (normalizer)	AF143208

### Amylase assay

Amylase activity was measured according to the method used in our previous study^19^ with a few modifications. Fresh cotyledons were homogenized in 50 mM K-phosphate buffer (pH 6.8) immediately after cultivation and the supernatant was used for amylase assay after being centrifuged. A mixture, containing 0.5 mL 2.5% starch solution, 0.3 mL 0.1 M sodium acetate buffer (pH 5.5), and 0.1 mL Milli Q water reacted with 0.2 mL of plant extracts at 55 °C for 5 min. The reaction was stopped by the addition of 0.5 mL 1 M HCl, and 0.2 mL aliquot of this mixture was diluted with distilled water to 10 mL, including 0.1 mL 1 M HCl and 0.1 mL 0.2% iodine solution. A blank was prepared by adding the plant extracts after the addition of HCl stopped the reaction. The absorbance of the solution was measured at 610 nm using a spectrometer (U3900, Hitachi, Japan).

### Statistical analysis

Thirty seedlings collected from at least four entirely independent experiments were used for growth measurements. Ten samples collected from at least three times entirely independent experiments were used for gene expression and α-amylase assay. We used the *t*-test to examine the difference between the control and clinorotation, and significant difference was accepted at *p* < 0.05. All data were represented as means ± standard error.

### Reporting summary

Further information on research design is available in the Nature Research Reporting Summary linked to this article.

## Supplementary information

Reporting Summary

## Data Availability

The datasets generated during and/or analyzed during the current study are available from the corresponding author on reasonable request.
